# De Novo Herpes Simplex Virus VP16 Expression Gates a Dynamic Programmatic Transition and Sets the Latent/Lytic Balance during Acute Infection in Trigeminal Ganglia

**DOI:** 10.1371/journal.ppat.1005877

**Published:** 2016-09-08

**Authors:** Nancy M. Sawtell, Richard L. Thompson

**Affiliations:** 1 Department of Pediatrics, Division of Infectious Diseases, Cincinnati Children’s Hospital Medical Center, Cincinnati, Ohio, United States of America; 2 Department of Molecular Genetics, Microbiology, and Biochemistry, University of Cincinnati School of Medicine, Cincinnati, Ohio, United States of America; Princeton University, UNITED STATES

## Abstract

The life long relationship between herpes simplex virus and its host hinges on the ability of the virus to aggressively replicate in epithelial cells at the site of infection and transport into the nervous system through axons innervating the infection site. Interaction between the virus and the sensory neuron represents a pivot point where largely unknown mechanisms lead to a latent or a lytic infection in the neuron. Regulation at this pivot point is critical for balancing two objectives, efficient widespread seeding of the nervous system and host survival. By combining genetic and in vivo in approaches, our studies reveal that the balance between latent and lytic programs is a process occurring early in the trigeminal ganglion. Unexpectedly, activation of the latent program precedes entry into the lytic program by 12 -14hrs. Importantly, at the individual neuronal level, the lytic program begins as a transition out of this acute stage latent program and this escape from the default latent program is regulated by de novo VP16 expression. Our findings support a model in which regulated de novo VP16 expression in the neuron mediates entry into the lytic cycle during the earliest stages of virus infection in vivo. These findings support the hypothesis that the loose association of VP16 with the viral tegument combined with sensory axon length and transport mechanisms serve to limit arrival of virion associated VP16 into neuronal nuclei favoring latency. Further, our findings point to specialized features of the VP16 promoter that control the de novo expression of VP16 in neurons and this regulation is a key component in setting the balance between lytic and latent infections in the nervous system.

## Author Summary

Herpes simplex virus remains a significant human pathogen associated with extensive acute and chronic disease in humans worldwide. The virus invades the peripheral and central nervous systems where it replicates but also establishes life-long latent infections in neurons. Two distinct viral transcriptional programs support these distinct lifestyles, but how entry into either the lytic or latent programs is regulated in the neuron is not understood. This process is fundamentally important to a virus with the capacity to be extremely virulent, in balancing two objectives, efficient widespread seeding of the nervous system and host survival. In this report, we provide new insight into this regulation and data that support a novel model in which virus transported into the neuron from the body surface enters the latent program by default. In a subset of these, there is a transition into the lytic cycle, which requires VP16 transactivation and is gated by a region in the VP16 promoter. Thus, HSV takes advantage of the anatomy and axonal transport systems in sensory neurons so that VP16 is left behind and latency is favored, while features of the VP16 promoter insure adequate virus spread in the nervous system and maximized latent infections.

## Introduction

Herpes simplex virus (HSV) remains a significant human pathogen associated with extensive acute and chronic disease in humans worldwide. Despite safe and effective antiviral treatment for limiting viral replication, the numbers of infected individuals continues to increase. The expansive reservoir of HSV genetic information is maintained unaffected by current antivirals within the nervous system of every infected individual. This reservoir of latent viral genomes fuels transmission, sporadically giving rise to infectious virus shed to surface sites [1]. Understanding how the virus establishes latent infections is an important goal for the development of new treatment strategies. Although progress has been made, the core mechanism(s) underlying the latent/lytic balance are yet to be defined.

The HSV life cycle is complex, interacting in a series of distinct and temporally shifting host cellular environments. Infection of a host at a mucosal surface and pursuant replication results in viral invasion of the innervating sensory nerve termini and transport to the sensory neurons in the ganglion. Viral replication occurs in the ganglion and at the periphery but is resolved within about 10 days. This acute stage of HSV infection is followed by a lifelong latent infection, primarily marked by the absence of signs of infection. Thus lytic and latent viral programs appear to be temporally distinct features of the viral life cycle. However, following the identification of the latent program promoter (LATp) [2] and generation of viruses carrying a LATp reporter gene [3], it became evident that during the acute stage of infection in the TG, both viral programs were represented in the population of infected neurons [3–5]. These findings revealed that viral program selection in the TG neuron was more complex and regulated in ways that were not understood. Several hypotheses have been proposed to explain this dichotomy, including neuronal type [6], transition from some lytic gene expression into the latent program [7], and alternative regulation of gene expression [8]. However, while progress has been made, the mechanism underlying this complexity remains unclear. Recent findings indicate that the virion transactivator protein, VP16, may play a pivotal role in these processes [9–11].

VP16 is an essential viral tegument protein required for virion morphogenesis [12]. In infected cell cultures VP16 also provides an important role in initiating the viral lytic cycle at low multiplicity of infection (moi), increasing plaquing efficiency 10–1000 fold in a cell type dependent manner [13–15]. VP16 has a potent carboxy-terminal acidic activation domain and an upstream core domain region that interacts with host cell proteins HCF and Oct-1 to form a VP16 induced complex (VIC) [16–18] (for review see [19]). These host cell partners are required for binding of the VIC to the TAATGARAT motifs unique to 5 viral immediate early (IE) gene promoters [20–25]. The possibility that latency in neurons is linked to the regulation of VP16 function, either through its absence (e.g. VP16 does not reach the neuronal nucleus or there is strict nonnuclear compartmentalization of essential co-activators), or its conversion from an activator to a repressor through altered neuronal cofactor interactions (e.g. HCF-2, Oct-2 or Brn3) have been proposed [19,25–33]. In an influential study, attempts to perturb the establishment of latent infections utilizing strategies to induce VP16 expression in infected TG failed [26], and reactivation (in an ex vivo setting) was not dependent upon VP16 transactivation function [27,34–36], limiting enthusiasm for the hypothesis that VP16 functioned as a central regulator in latency and reactivation in vivo. At this time, an alternate viral protein, IE gene ICP0, became the focus of intense investigation as possibly initiating reactivation. The ability of ICP0 to induce modifications of the histone codes on quiescent/latent viral genomes from those associated with repression to those associated with derepression and induce the transcription of reporter promoters in tissue culture models make it an attractive candidate (recently reviewed in [37–39].

In more recent studies, two distinct VP16 transactivation deficient mutants failed to exit latency in vivo, both blocked at a very early stage prior to viral protein synthesis [11,40]. In addition, evidence that de novo expression of VP16 may be required for viral replication in neurons during the acute stage of infection has also been reported [11]. In cultured neurons strong evidence that VP16 dissociates from the nucleocapsid prior to retrograde transport into the nucleus favoring quiescence (latency) has been published [41,42] although alternate mechanisms of retrograde transport of VP16 occur that can induce viral replication [43,44].

The design of experiments to test the functions of VP16, ICP0 or other factors in the latent/lytic decision is not straightforward. Infection of neurons in the TG is highly asynchronous, with new infections and superinfections occurring in the TG from virus transported from the surface over multiple days. Thus studies of the temporal expression patterns in neurons are difficult to interpret. Already by 32–36 hrs pi, infectious virus is detectable in the TG [45,46] and when examined at 3 days pi, neurons undergoing productive lytic infection were found temporally and spatially distributed among neurons in which the viral latent program was activated [3,5,47,48]. At very early times pi, extremely few trigeminal ganglion (TG) neurons are infected. For these reasons classic single cell based measures such as immunohistochemistry and in situ hybridization on tissue sections, or bulk analyses such as quantitative reverse transcription PCR, proteomic approaches and RNAseq cannot adequately address these important questions.

In this study we sought to overcome these difficulties by employing viral mutants expressing reporter proteins from viral promoters of diverse kinetic classes or viral transcriptional regulators expressed from the LAT promoter, combined with whole ganglion histochemical and immunohistochemical techniques. This permits the interrogation of the earliest events in TG neurons infected from the body surface in the context of essentially wild type virus infection at the single neuron level. In addition whether select viral proteins are sufficient to alter the balance between latent and lytic infection in neurons in vivo can be determined.

Our findings reveal that the balance between latent and lytic programs in individual neurons is (i) observable, (ii) that entry into the latent program is the default (occurs first), (iii) the lytic cycle begins as a transition out of this early latent program, (iv) VP16 can play a dominant role in this transition, and (v) the VP16 promoter contains elements dispensable in cultured cells and corneas, but required for efficient transition out of the default latent program and productive lytic infection in neurons. Importantly, these findings reveal a previously unrecognized transition occurring in individual neurons. This transition from latent into lytic programs is gated, at least in part, by de novo VP16 expression and emphasizes the importance of the novel regulation of this gene during acute infection of sensory neurons in vivo.

## Results

### Strategy for testing the outcome of de novo expression of VP16 in trigeminal ganglion (TG) neurons

We exploited the properties of the basal latency associated transcript promoter (LATp) to express selected open reading frames from the viral genome. Prior studies demonstrated that the (LATp) (driving expression of the E. coli beta galactosidase gene (LacZ)) is silenced in the context of infection in cultured cells [49–51]. However, in the mouse corneal model, as early as day 3 pi, this promoter is active in a population of trigeminal ganglion (TG) neurons which are largely devoid of viral proteins [3,48,52,53]. Importantly, this promoter contains neuronal specific elements, and an ICP4 binding site that down regulates its activity very early in the lytic cycle [49–51]. These features provide for a viral platform in which an open reading frame driven by the LATp would fail to express during lytic infection (and thus would not alter the normal lytic cycle) but would express de novo (in the absence of an ongoing lytic cycle) in a population of TG neurons. Since our focus in these studies is neurons that have entered the latent program during the acute stage of infection (in contrast to the common use of “latent” at times >30days pi), we will designate this as acute stage latent program to avoid confusion.

### Design of viral mutants and functional assessment of constructs

**Fig 1:**
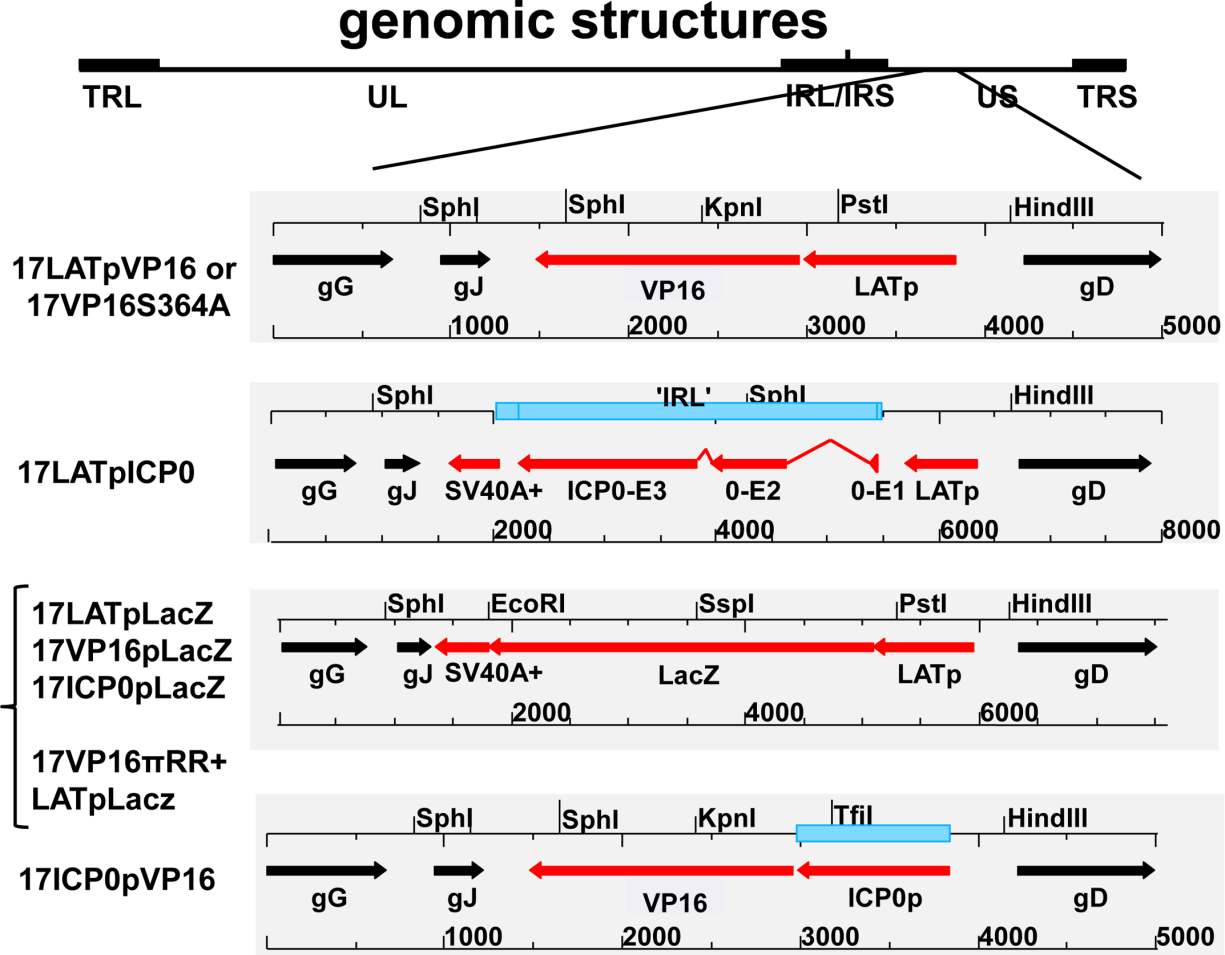
Mutants generated for these studies. Schematic representations of the genomic structures of all new viral mutants generated for these studies. Open reading frames and promoter sequences are indicated by filled arrows (Black = genes resident in the locus, Red = transgene). Selected restriction endonuclease sites and base pair lengths are indicated above and below each diagram. All transgene expression cassettes were inserted after base pair 138047 in the HSV-1 strain 17syn+ genome. Insertion at this site does not perturb expression of any viral genes [11,54].

The design of the viral mutants is shown schematically in Fig 1. Importantly, placement of the expression cassettes after base pair 138047 in the intergenic region between glycoprotein J (gJ) and gD leaves all known wild type proteins intact and does not alter the transcription of either the gJ or gD genes [11]. Since expression from the LAT promoter is silenced during lytic infection [2,51], LacZ, the second copy of VP16, or the third copy of ICP0 driven by LATp would be unexpressed. However, it is possible that the LATp is leaky in neurons and that the effects of a second copy of VP16 expressed early in the lytic cycle could confound the interpretation of in vivo studies. Therefore, an additional control mutant containing a second copy of VP16 driven by the ICP0 promoter was constructed.

### Structural and in vitro phenotypic characterization of constructs and viral mutants

The ability of the proteins expressed from the mutagenesis constructs to enhance expression of relevant promoters in reporter assays was confirmed by transient co-transfection assays (S1A Fig). At least three independently derived viral mutants of each type were generated as detailed previously [11,55,56] and in methods. In some cases genomically restored isolates were also constructed and tested to confirm that the altered phenotypes displayed by the mutants were the result of the intended mutation. RFLP analysis of the mutants revealed the predicted genomic structures (for example see S2A and S3A Figs) and sequence analysis demonstrated correct insertion of the mutation cassette into the viral genome. Biological characterization was performed in rabbit skin cell (RSC) cultures to assess (i) replicative capacity using multistep replication kinetics, (ii) levels of VP16 or ICP0 mRNA and protein in the context of 17LATpVP16 and 17LATpICP0 infection, and (iii) levels of VP16 mRNA and protein in the context of ICP0pVP16 infection. The mutants employed are genotypically as expected, and as shown (S1B–S1E Fig) are replication competent, and express the inserted transgenes with the expected kinetics in RSC cultures.

### In vivo characterization: VP16 but not ICP0 driven by the LATp results in a temporal shift in the production of infectious virus in TG

**Fig 2:**
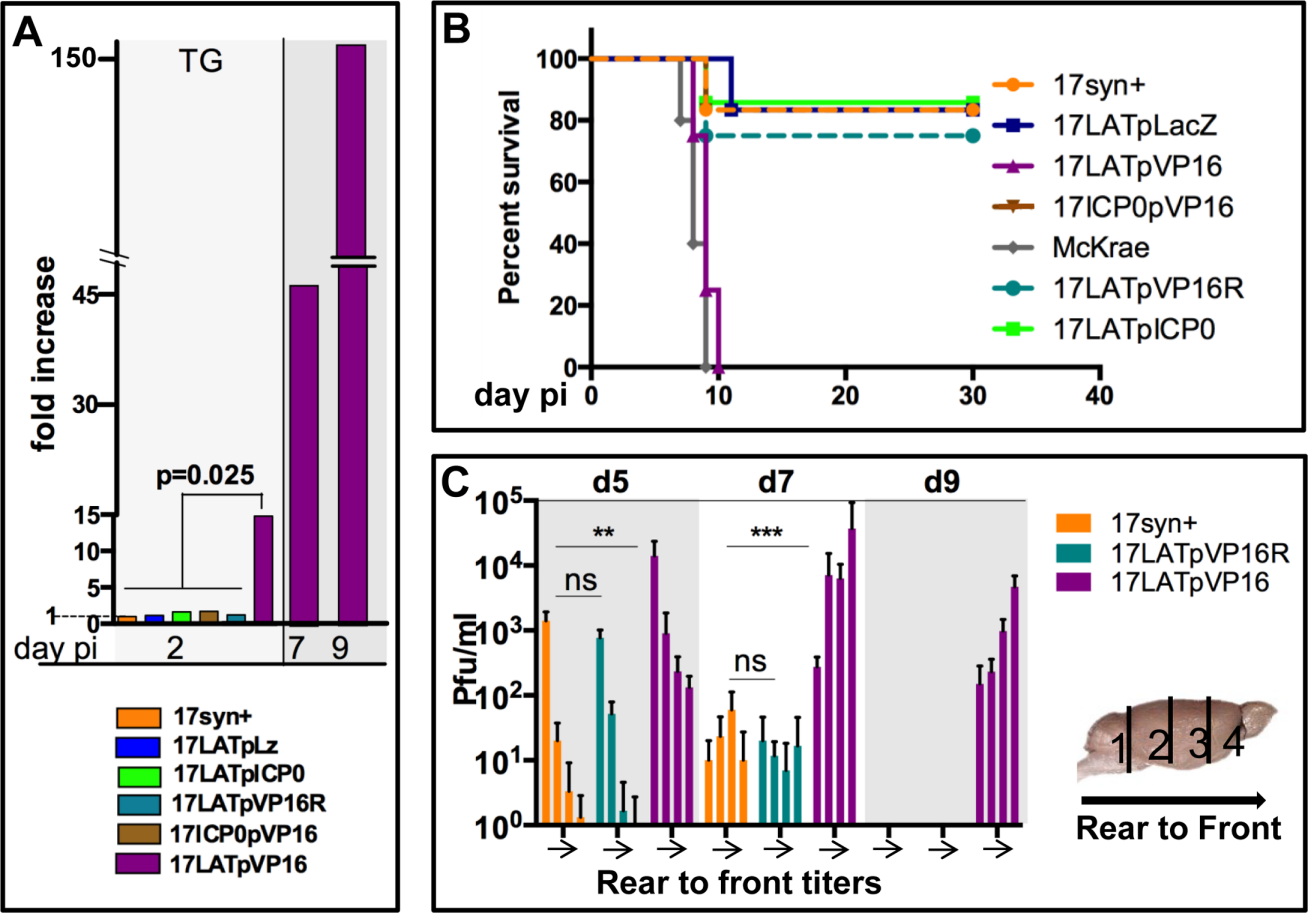
In vivo phenotypes of mutants expressing extra copies of ICP0 and VP16. A) Viral replication in trigeminal ganglia (TG). Outbred Swiss Webster mice were infected via both corneas with 1x10^5^ pfu of the indicated virus isolates. Tissues from three mice per viral mutant per time point were harvested and analyzed for infectious virus titers. Bars represent the fold increase in titer relative to the parental strain 17syn+. Viral titers appear in the text. B) Survival was determined in groups of mice infected on the eyes with 1x10^5^ pfu. The color code is the same as that in Fig 2A with the addition of a gray colored line which represents mice infected with the wild type strain McKrae (a virulent laboratory strain). C) Replication and spread of virus within the central nervous system was assessed by examining virus titers in four roughly equal divisions as depicted below the graphs. Viral titers detected on days 5, 7 and 9 pi in the CNS of three mice from each group are shown. 17LATpVP16R is a genomically restored isolate in which the VP16 transgene in the 17LATpVP16 mutant was removed (detailed in methods). Each bar represents the average of the amount of virus found in each of the brain sections. There was no significant difference between 17syn+ and 17LATpVP16R infected brain regions (bar labeled NS for not significant). There was significantly more virus detected in all four brain sections infected with 17LATpVP16 compared to 17syn+ and 17LATpVP16R (bar labeled ** is p<0.05; labeled *** is p<0.001). Both Fisher’s exact test and unpaired Student’s t-test were employed.

Our prediction was that if VP16 and/or ICP0 could drive entry into the lytic cycle from the activated LATp, a significant increase in viral titer in the TG would be observed. Three independent isolates of each virus were analyzed. One of the 17LATpVP16 mutants (isolate 17LATpVP16 4AA1) was restored to wild type sequence (see methods) to further test that the phenotypic changes detected were not the result of some unknown second site mutation. Significantly, although independently derived, the phenotypes were consistent for a given set of mutants. Infection of mice with 1x10^5^ pfu of 17syn+, 17LATpICP0, 17LATpVP16, 17LATpVP16R, or 17LATpLacZ yielded similar infectious titers in the eyes through day 4 pi (day 2 pi, 1.8x10^4^ ± 6.6x10^3^ to 2.8x10^4^ ± 5.1x10^3^, p = 0.76 ANOVA; day 4 pi, 5.6x10^4^ ± 4.6x10^3^ to 6.4x10^4^ ± 4.3x10^3^, p = 0.41, ANOVA). 15-fold more infectious virus in TG of 17LATpVP16-infected mice was observed already on 2 dpi, (17syn+, 17LATpICP0, 17LATpVP16R, and 17LATpLacZ ranged from 146.7 ± 55.8 to 234.7 ± 76.4, average 195.3 ± 69.55 vs 2,967 ± 1041 pfu/ml for 17LATpVP16). On day 4, when virus titers peak in the TG, the differences in titers among the viruses varied by less than 1.7 fold, ranging, 6x10^4^ to 1x10^5^. However, on 7 dpi, when titers are normally declining in the TG, there was a 45-fold difference (range 183.3 ± 104.1 to 329.94 ± 122.3, average 211.7 ± 156.3 vs. 9,540 ±1,323 pfu/ml) and >1,000-fold (range 0 to 10 vs. 800 to 10,000 pfu/ml) on day 9 (Fig 2A). All of the mutants tested with the exception of the 17LATpVP16 mutants exhibited virulence profiles similar to that of the parental strain 17syn+ with 75% or more of the mice surviving inoculation with 1x10^5^ pfu (Fig 2B). In contrast, all of the 17LATpVP16 isolates tested were significantly more virulent than the parental strain 17syn+ (Fig 2B and Table 1 below).

**Table 1:** Analysis of Virulence Phenotypes.

Virus Isolate	Virulence
17syn+	>10^6^ ^a^
17LATpICP0	>10^6^
17LATpLacZ	>10^6^
17ICP0pVP16	>10^6^
17LATpVP16	5.2 to 7.3 x10^3^ ^b^
17LATpVP16R	>10^6^ ^C^
McKrae	<10^3^

a) No attempt was made to reach endpoints above 10^6^ or below 10^3^ PFU.

b) Range displayed by three independently derived mutants.

c) Two independently derived genomically repaired (rescued) variants of 17LATpVP16.

### De novo expression of VP16 is correlated with increased viral replication in the central nervous system

To determine whether the increased virulence of 17LATpVP16 infection correlated with increased viral replication in the CNS viral replication in the central nervous system CNS was quantified over time. Groups of male Swiss Webster mice were infected with 1x10^5^ pfu on the corneal surface with 17syn+, 17LATpVP16, or 17LATpVP16R. As early as 2 days pi, 100% (4/4) of brains from mice infected with 17LATpVP16 contained low levels of infectious virus, ranging from 13–150 pfu which increased to a mean of 2.5x10^3^ (range: 900–5000) by day 4 pi. In contrast, virus was not detected in the brains harvested from either 17syn+ or 17LATpVP16R infected mice on day 2 and on day 4 pi, a mean of 7 pfu (range: 0–20) was detected, a difference of ~300 fold (p<0.0001, ANOVA). As shown in Fig 2C, infectious virus titers in all portions of the brains from 17LATpVP16 infected mice (n = 4) were greater than those detected in 17syn+ (n = 3) or 17LATpVP16R (n = 4) infected brains by 10 to >1000 fold. At times when replication in the CNS was no longer detected in either 17syn+ or 17LATpVP16R infected brains, replication continued in 17LATpVP16 infected mice with more than 1000 pfu present in the front portions of the brain. Thus the increase in mortality of 17LATpVP16 directly correlated with earlier, increased, and prolonged viral replication in the CNS (Fig 2C).

### De novo expression of VP16 from the LAT promoter increases virulence in both susceptible and resistant genetic backgrounds

Virulence was evaluated by infecting groups of male Swiss Webster mice on corneas with serial dilutions of 17syn+, the viral mutants, 17LATpVP16R, or wild type strain Mckrae. This strain was included as a benchmark of “natural virulence” observable among unmodified HSV isolates. Virulence data is summarized in Table 1.

While the increase in virulence displayed by the 17LATpVP16 mutants was significant, these mutants did not exceed the virulence of the laboratory strain McKrae and thus remained within the range of virulence naturally occurring among human isolates. We also tested the effect LATpVP16 expression in the highly resistant C57BL/6J background. Groups (3–5 mice/group) of age and sex matched adult C57BL/6J mice were infected on scarified corneas with 1x10^6^ pfu of either 17syn+, 17LATpLacZ, 17LATpVP16, or McKrae. All of the mice infected with 17syn+ or 17LATpLacZ survived, in contrast, none of those infected with either 17LATpVP16 (0/4) or McKrae (0/5) survived.

### 17LATpVP16 phenotypes require VP16 transactivation function

To test the importance of the transactivation function of VP16 to the observed phenotypes, we generated an HSV-1 mutant that expresses a second copy of a transactivation null VP16 from the LATp in the gJ locus (17LATpVP16/Y364A). This VP16 ORF contains a single amino acid change of tyrosine to alanine at position 364 that disrupts the ability of VP16 to bind to its co-activator protein HCF-1 [57,58]. Consistent with previous reports, the ability of the VP16 with the Y364A mutation to transactivate the ICP0 promoter in transient transfection assays was 15 fold reduced compared to WT VP16 and not different than the empty vector control in cultured cells [59,60]. Characterization of the genomic structure of six independently derived isolates revealed insertion in the gJ locus but no other perturbations (S2A Fig). The multi-step replication of the mutants in RSC in vitro was not significantly different than the parental strain (S2B Fig).

Groups of mice were infected with 1x10^5^ pfu each of the three independently derived isolates of 17LATpVP16/Y364A or wild type 17syn+. Survival was evaluated and at various times pi tissues were collected and analyzed for infectious virus. Neither survival (17LATpVP16/Y364A, 8/10; 17syn+, 7/9) nor replication (17LATpVP16/Y364A, d4pi TG, 5.7x10^4^ ± 6.8x10^3^; 17syn+, d4pi TG, 7.7x10^4^ ± 1.3x10^4^) were different, p = 0.25, Student’s t-test) demonstrating that the transactivation function of VP16 was required for the 17LATpVP16 enhanced virulence phenotype.

### The 17LATpICP0 mutant viruses were genomically stable and the third copy of ICP0 retained its ability to transactivate

The finding that de novo expression of ICP0 from the LATp did not alter viral replication in the TG was somewhat unexpected. There are several lines of evidence supporting a role for ICP0 in either enhancing [61,62] or initiating reactivation from latency [61–70]. However, in studies discriminating between roles in initiation or in progression, ICP0 was not required for the efficient initiation of reactivation from latency, but essential for infectious virus production in reactivating neurons in vivo or in the setting of quiescent infection in cultured cells [54,71].

To test for the possibility that gross genomic rearrangements of ICP0 might occur during replication in vivo, three independently derived isolates of the 17LATpICP0 mutants were examined. Virus was recovered from the eyes and TG from mice on day 5 pi (three mice per isolate). Viral DNA was examined by Southern blot for RFLPs. Rearrangements were not detected in any of the eighteen DNA samples derived from acutely infected tissues (S3A Fig). Viral DNA isolated from latently infected TG following explant reactivation [72] was also examined. In this case, nine virus samples were obtained from groups of three mice latently infected with each of one of the three 17LATpICP0 isolates. No evidence of genomic instability was detected in any of the samples (S3A Fig). Finally, the LATpICP0 gene cassette was cloned from the recovered viruses and employed in transient transfection assays to test the function of the recovered ICP0 genes. Without exception, the recovered LATpICP0 gene cassettes transactivated a target plasmid similarly to the original clone employed to produce the viruses (S3B Fig).

### The failure of ICP0 driven by the LAT promoter to initiate viral replication was not due to silencing by latency related RNAs

The LAT locus expresses virally encoded large and small non-coding RNAs with the potential to interfere with ICP0 protein expression. These include the stable LAT introns that are partially antisense to the ICP0 mRNA [73] and viral miRNAs that may target the ICP0 mRNA [74–77]. Because these RNAs could be expressed in neurons in which the LAT promoter is active and might interfere with the expression of ICP0 protein in these neurons [54,78], the LATpICP0 cassette was recombined into our prototypical LAT null mutant 17AH (in which none of these RNAs are expressed from the LAT promoter [79] [80]) to generate 17AHLATpICP0 mutants. Groups of mice were infected with three independently derived isolates of 17AHLATpICP0 and a control virus that expresses LacZ from the LATp in the same locus in the 17AH background. Viral replication in eyes and TG were quantified on days 2,4,6,8, and 10 pi. No difference in viral replication in eyes or TG was observed between the mutants and control virus. Titers on day 4 pi in 17AHLATpICP0 infected eyes ranged from 4.4x10^4^ ± 3.2x10^3^ to 4.9x10^4^± 5.1x10^3^ pfu/ml, values not different from each other or from 17AHLATpLacZ infected eyes (4.6x10^4^± 1.1x10^3^ pfu/ml) (p = 0.64, ANOVA). Viral titers in TG were also not different, peaking on day 4 pi in both groups at 1.4x10^4^ ± 4.7x10^3^ to 3.3x10^4^± 2.0x10^3^ pfu/ml and 2.9x10^4^ ± 4.1x10^3^ pfu/ml in TG from 17AHLATpICP0 and 17AHLATpLacZ infected animals, respectively. No increase in virulence was observed in mice infected with the 17AHLATpICP0 mutants compared to 17AHLATpLacZ.

### Elevated viral titers in TG infected with 17LATpVP16 mutants at early times pi correlates with more neurons entering into the lytic transcriptional program

**Fig 3:**
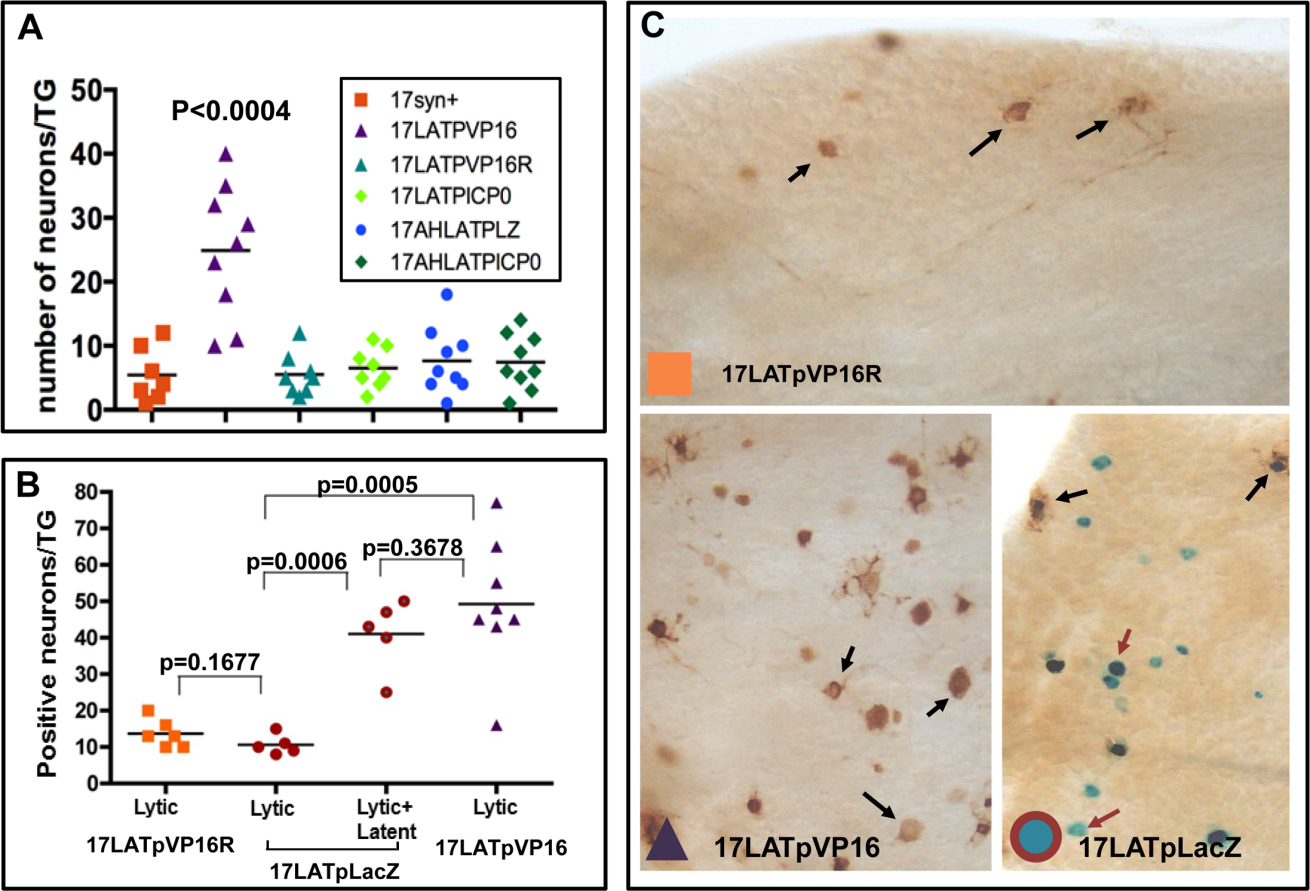
Quantification of the number of neurons expressing the lytic and/or the latent transcriptional program markers. A) Quantification of neurons positive for viral protein at 40 hrs pi. This experiment included 17LATpVP16R, the genomic rescue of 17LATpVP16, as well as 17LATpICP0 and two mutants made on the LAT null background parent 17AH (17AHLATpLacZ and 17AHLATpICP0). There was no significant difference in the number of neurons positive for viral proteins [11] in whole TG except for the TG infected with 17LATpVP16, which contained significantly more positive neurons (p<0.0004, Fisher’s exact test). B) Quantification of neurons positive for viral protein, LATp activity, or both at 46 hrs pi. Each point represents the number of positive neurons in a TG. C) TG were fixed and processed for the simultaneous detection of b-gal activity (blue neurons:red arrows) and viral protein expression (brown neurons:black arrows). Shown are representative photomicrographs of whole ganglia at the same magnification. The number of neurons in TG expressing either viral proteins (lytic), LacZ from the LAT promoter (“latent”), or both (lytic+“latent”) were enumerated (shown in B).

Because a significant rise in viral titer was observed in the TG of mice infected with 17LATpVP16 mutants by 48 hrs pi (Fig 2A) we examined viral protein expression in TG neurons at 40 hrs pi, a time at which viral spread is minimal in wild type infected TG. Groups of mice were infected with 1x10^5^ pfu of either 17syn+, 17LATpVP16, 17LATpVP16R, 17LATpICP0, 17AHLATpICP0, or 17AHLATpLacZ. At 40 hrs pi, eyes and TG were harvested and infectious virus was quantified in eye homogenates and whole ganglia were stained for HSV proteins and positive neurons counted (Fig 3A). There was no significant difference in the amount of infectious virus detected in eyes with values ranging from 1.6x10^4^ to 2.7x10^4^ pfu/ml and only TG infected with17LATpVP16 had significantly more positive neurons (see Fig 3A, p<0.0004, ANOVA). Thus prior to day 2, the expression of VP16 from LATp increases the number of TG neurons entering the lytic program by 6 fold. In addition, there is no significant difference in the number of lytic program neurons between 17LATpICP0 and 17AHLATpCP0.

A variation of this experiment using the LacZ gene in the 17LATpLacZ to determine the relationship between the number of neurons LATp positive with the number of neurons expressing viral protein was performed. Groups of mice were infected with 17LATpVP16, 17LATpLacZ, or 17syn+. Eyes and TG were harvested at 46 hrs pi. Viral titers in eyes were not different among the groups. TG were processed for the dual detection of b-gal activity and viral protein expression as previously described [11,54]. The 17LATpVP16 infected ganglia contained ~4 fold more neurons expressing viral protein compared to 17syn+, or the 17LATpLacZ mutant (49.3 ± 6.3 vs. 13.7 ± 1.6 or 11.4 ± 1.8, p<0.0005, ANOVA Fig 3B). Importantly, in 17LATpLacZ infected TG, the number of combined LATp and lytic positive neurons was similar to the total number of neurons positive for viral protein in 17LATpVP16 infected TG (42 and 49) (Fig 3B). Representative photomicrographs of TG are shown in Fig 3C. Positive neurons in all groups were distributed as isolated neurons indicating that these neurons represent primary infections and not secondary spread. These types of experiments were repeated several times with consistent outcomes.

### The latent program precedes lytic program activation in TG neurons early following infection from the periphery

The preceding studies demonstrate that VP16 expressed de novo from the LATp in TG neurons disrupts the acute stage latent program and that the first phenotypic evidence for this occurs earlier than 48 hrs pi. However, what underlies the “decision” to enter the acute stage latent or lytic program during a wild type infection remains an open question. Mapping the relative numbers of neurons and timing pi that either the latent or lytic program becomes active in neurons could be informative. We utilized a set of viral promoter LacZ reporter mutants (representing immediate early, late, and latent transcription) (Fig 1) to determine the number of neurons and activation order of the lytic vs. latent transcriptional programs in neurons in vivo at early times pi. As shown here for 17LATpLacZ (Figs 2A and 3C) and previously for 17VP16pLacZ and 17ICP0pLacZ [11,54,78], the viruses employed replicated equivalently in vivo.

**Fig 4:**
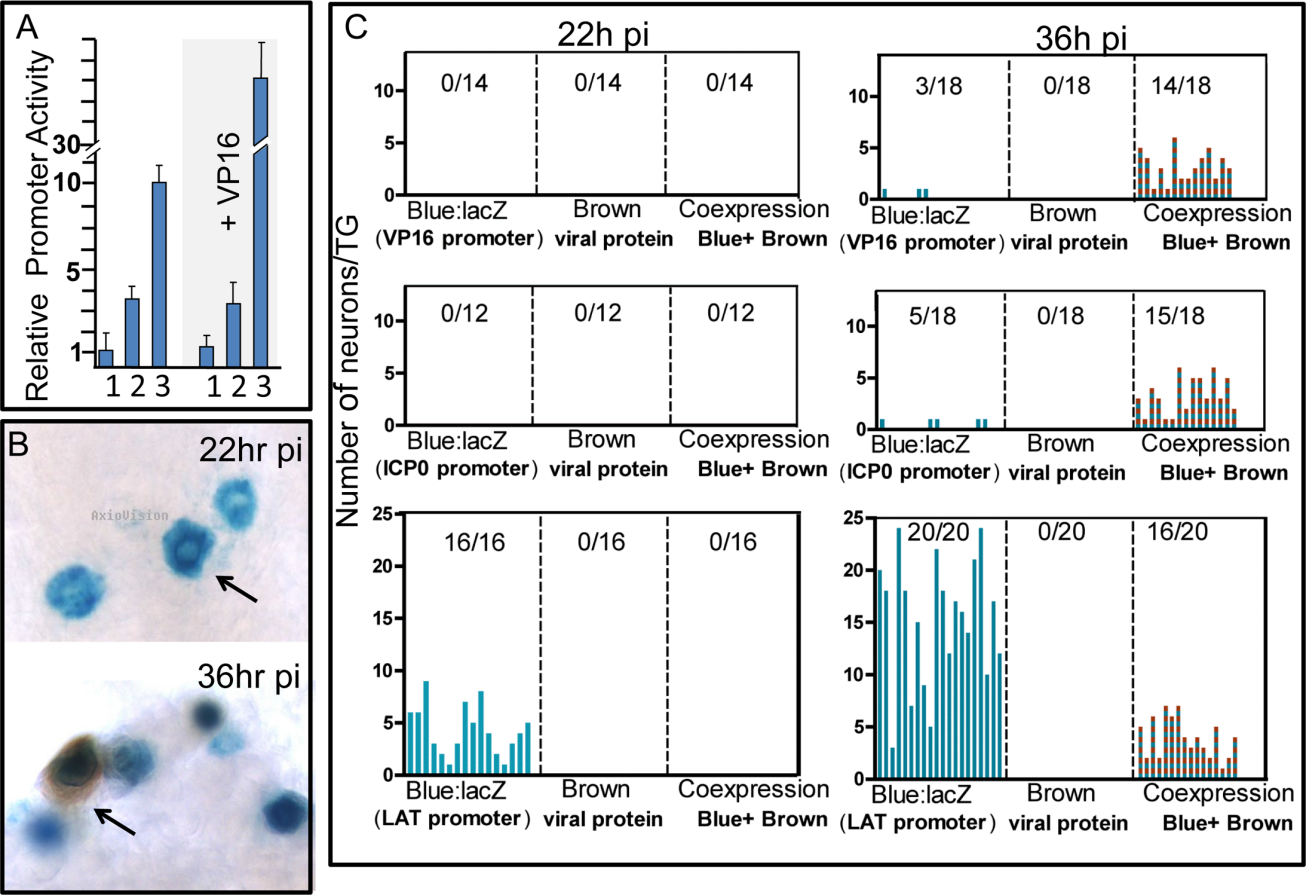
Quantification of viral lytic and acute stage latency gene transcription in individual neurons at very early times pi. A. Relative promoter strength was assayed by transfection of rabbit skin cells with the constructs employed to generate the viral promoter/reporter mutants and analysis of the amount of b-gal determined with a CPRG assay as detailed in methods. Transfection efficiencies were normalized by including a renilla luciferase plasmid in the assay. Lane 1 = LATpLacZ, 2 = VP16pLacZ, 3 = ICP0pLacZ. The LAT promoter was the weakest and the levels of expression of this construct were set to one. Where indicated, a VP16 expressing plasmid was included in the transfections. Each bar represents the average of 3 transfection experiments of 3 wells each. B. Photomicrographs of TG from mice infected with17LATpLacZ processed for LATp activity and viral protein expression. At 22 hrs pi, expression was restricted to the LATp. A 36 hrs, viral proteins are expressed but restricted to a subset of neurons that are marked by LATp activity. C. At the indicated times pi, eyes and TG were harvested. Unsectioned TG were processed for the histochemical detection of b-gal activity and IHC detection of viral proteins as detailed previously [4,11,48,54,78,81]. Bars represent the number of neurons positive in an individual TG. The number of TG positive over the number tested in each group is indicated. All TG tested from mice infected with 17LATpLacZ contained one or more neurons positive for b-gal activity at 22 hrs pi, whereas no TG infected with the lytic stage promoter reporter viruses were positive (p≤0.0001, ANOVA). At 36 hrs pi a subset of ganglia examined contained neurons positive for both b-gal and viral protein. The number of ganglia positive and the number of positive neurons in the TG was not different between groups (p≥0.5, ANOVA). Importantly, all neurons positive for viral protein were also positive for LAT promoter activity in the 17LATpLacZ group.

A potential complicating factor is relative promoter strength since “timing” of expression might be influenced by expression if there were very large differences in promoter activity. We analyzed relative promoter strengths in cultured cells with transient assays utilizing the promoter/reporter constructs that were employed to generate promoter/reporter viruses. As previously reported, the LacZ ORF was cloned behind the immediate early ICP0 gene promoter (ICP0p) [54,78], the leaky late UL48 gene promoter (VP16p) [11] or the basal LATp as detailed in methods. As shown in Fig 4A the ICP0p was slightly stronger than the VP16p promoter and both lytic phase promoters were stronger than the basal LATp. One caveat is that VP16 protein would be carried into the cell in the virion tegument. To mimic this condition an additional plasmid expressing VP16 from the human CMV IE gene promoter was added to the transfections. The order of the relative strengths of the promoters remained the same (Fig 4A). The addition of VP16 increased expression from the ICP0 promoter, but had no significant effect on the VP16p or LATp. In vivo in TG neurons findings were similar in that it required more time for blue color to develop in TG infected with 17LATpLacZ than did TG infected with either 17ICP0pLacZ or 17VP16pLacZ. While only semi-quantitative this is consistent with the relative activity of the promoters in transient assays. As seen in Fig 4, LacZ expression from the lytic promoters coincided with detection of viral proteins, suggesting the native and reporter promoters behaved similarly and that the moderate difference between the strengths of the ICP0 and VP16 promoters did not affect detection on the time scale employed here.

Preliminary experiments to determine the earliest time that activity could be detected from these promoters in the TG revealed that while no viral promoter activity was detected at 10 hrs pi, by 18 hrs pi, activity exclusively from the LATp was observed in rare neurons in a subset of TG. The global view afforded by the whole ganglia approach revealed that at these early time points, all viral activity was restricted to neurons (based on morphological criteria). In these experiments, mice were infected on corneas with 5x10^5^ pfu and groups of 20 TG were examined for each virus at each time point. As shown in Fig 4C, at 22 hrs post infection *all* trigeminal ganglia from mice infected with the 17LATpLacZ mutants contained multiple neurons positive for b-gal activity. In contrast, *none* of the ganglia from mice infected with the 17ICP0pLacZ or 17VP16pLacZ mutants contained b-gal positive neurons (p<0.0001, Fisher’s exact test). The TG were subsequently processed for the detection of viral proteins as previously detailed [54,82]. Viral protein expression was not detected in any of the ganglia at this early time (in contrast to 40–46 hrs, Fig 3C). Importantly, titers of virus in the eyes were not different among the groups, ranging from 1.2x10^5^ to 3.1x10^5^ pfu per pair of eyes at 22 hrs pi.

### Identification of a transition from the acute stage latent program into the lytic transcriptional program in individual TG neurons

We analyzed viral promoter and viral protein expression at times later than 22 hrs pi to determine (i) timing of lytic promoter activation, (ii) timing of viral protein expression, (iii) the relationship between lytic promoter activity and protein expression, and (iv) the relationship between acute stage latent neurons and viral protein expressing neurons. Viral protein could be detected in rare neurons as early as 32 hrs pi in a subset of TG from all infected groups. The results of analysis of TG from additional infected mice at 36 hours pi are shown in Fig 4C. The amount of virus detected in the eyes of these mice was not different between groups at the time of tissue harvest (p = 0.70, ANOVA). In mice infected with the 17LATpLacZ reporter mutant 20/20 (100%) of ganglia tested contained numerous neurons positive for only b-gal, and far more neurons were positive for b-gal alone than for viral proteins (Fig 4C, p<0.0001 Fisher’s exact test). A similar subset of ganglia contained neurons positive for viral proteins in all groups examined 16/20 (80%, 17LATpLacZ mutant), 14/18 (77%, 17VP16pLacZ mutant) and 15/18 (83%, 17ICP0pLacZ mutant) (p>0.72, Fisher’s exact test). Likewise there was no difference in the distribution and number of neurons positive for viral proteins in these ganglia (Fig 4C, p = 0.79, ANOVA). Thus, infection of TG was similar with all of the promoter/reporter mutants as determined by the number of neurons positive for viral protein expression. In every case in these TG, all neurons positive for viral proteins were also positive for b-gal.

In contrast, in TG infected with the 17LATpLacZ mutants, 3 to 4 fold more neurons were positive for b-gal than those expressing viral proteins. Of particular importance, *all* neurons positive for viral proteins in ganglia infected with 17LATpLacZ mutants were also positive for b-gal activity at this time. No viral proteins were detected in non-blue neurons in this group of TG. These findings offer compelling support for the idea that in sensory neurons infected from the body surface HSV first enters the latent transcriptional pathway and expresses the LAT locus. About 12–14 hrs later a portion of the LAT expressing neurons transition from this acute stage latent program into the lytic transcriptional pathway (the dual labeled neurons) while other neurons remain b-gal positive only (e.g. remain in the latent transcriptional program). These findings reveal an important feature of the early events in neuronal infection in vivo, namely that entry into the lytic cycle is gated through a transition from an early latent program.

### Mutations in the VP16 promoter with no effect on replication at the periphery or in cultured cells reduce replication in the TG by 100 fold and reduce transition from acute stage latent program into the lytic program 15 fold

**Fig 5:**
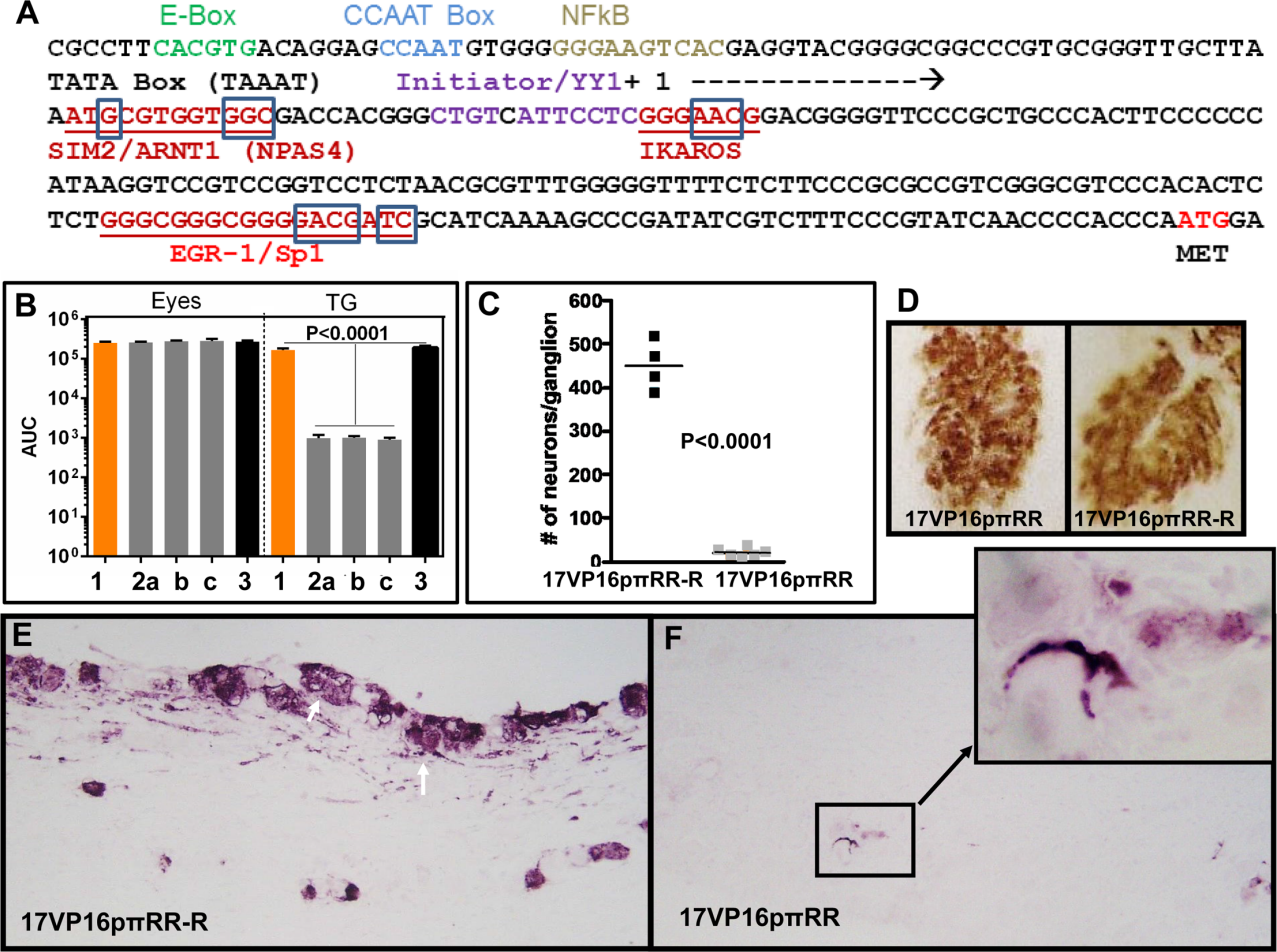
Entry into lytic infection in TG neurons is regulated by sequences in the VP16 promoter. A) Sequence of the proximal VP16 promoter. Putative regulatory sites identified previously [83,84] are color coded and labeled above the sequence. Potential sites known to confer reciprocal regulation to neuronal genes are underlined and in red. The boxed nucleotides were altered as detailed in methods. B) Replication kinetics on eyes and in TG on days 2 through 10 pi is shown as the area under the curve (AUC). Tissues from 3 mice were examined for each time point. 1 = 17syn+, 2a, b, c are three independently derived isolates of the VP16 promoter mutant 17VP16pπRR and 3 = the genomically restored isolate 17VP16pπRR-R. C) Mice were infected on the cornea with the VP16 promoter mutant 17VP16pπRR or its genomically restored isolate 17VP16pπRR-R. At 72 hrs pi tissues were harvested and processed for the whole tissue immunohistochemical detection of VP16 and the number of VP16 positive neurons enumerated. The data are shown as a scattergram with each point representing the number of positive neurons in an individual TG. The difference between the groups was significant (p<0.0001, Student’s t-test). D) Representative photomicrographs of corneas stained for VP16 protein which is seen as a brown precipitate in characteristic lesions on the corneal surface (arrows). E) Representative photomicrographs of sectioned trigeminal ganglia infected with 17VP16pπRR-R stained immunohistochemically for VP16 (purple precipitate). Numerous cells including neurons identifiable by their large size, morphology and axonal tracts are positive in the TG infected with 17VP16pπRR-R the genomically restored isolate which is not different than WT 17syn+ (white arrows). F) Rare VP16 positive cells in the 17VP16pπRR infected TG were detected and did not appear to be neurons. One such area is boxed and shown enlarged in an inset micrograph. This region appears to be a small focus of positive support cells.

We reported previously that while the VP5 promoter could substitute for the VP16 promoter in cultured cells and on the corneal surface, replication in the TG was impaired despite equivalent viral genomes feeding into the ganglion [11]. This suggested that unique regulatory information was embedded in the VP16 promoter. While detailed mapping of functional sites in this region is ongoing, a region of interest was identified near and downstream of the VP16 TATA box and one set of mutants generated has phenotypic properties similar to those observed when the VP5 promoter was used to drive VP16 expression ([11] and Fig 5). This mutant named 17VP16pπRR (for preIE regulatory region) contains 13 nucleotide changes clustered within three elements predicted to bind factors known to reciprocally regulate stress responsive neuronal genes (see methods, changed nucleotides are boxed in Fig 5A).

Three independently derived 17VP16pπRR mutants were generated as detailed in methods. All three isolates (i) express wild type VP16, (ii) exhibit no plaquing deficits, (i.e. are not hexamethylene bisacetamide responsive [13]), and (iii) replicate like wild type in cultured cells and on the corneal surface (Fig 5B). These outcomes confirm that VP16 is packaged into virions sufficient to support low moi infection indistinguishable from wt virus. However, these mutants are extremely defective for replication in TG (Fig 5B). At 3 days pi there were >100-fold fewer neurons positive for VP16 in TG infected with the promoter mutant than found in TG infected with wild type (Fig 5C). Eyes and TG infected with 17syn+, 17VP16pπRR, or 17VP16pπRR-R were examined for VP16 expression using whole and sectioned tissue immunohistochemical methods. The intensity and distribution of VP16 protein on corneas was not different between infection with 17VP16pπRR or 17VP16pπRR-R (Fig 5D) or 17syn+ but VP16 was not efficiently produced in TG (Fig 5E and 5F).

**Fig 6:**
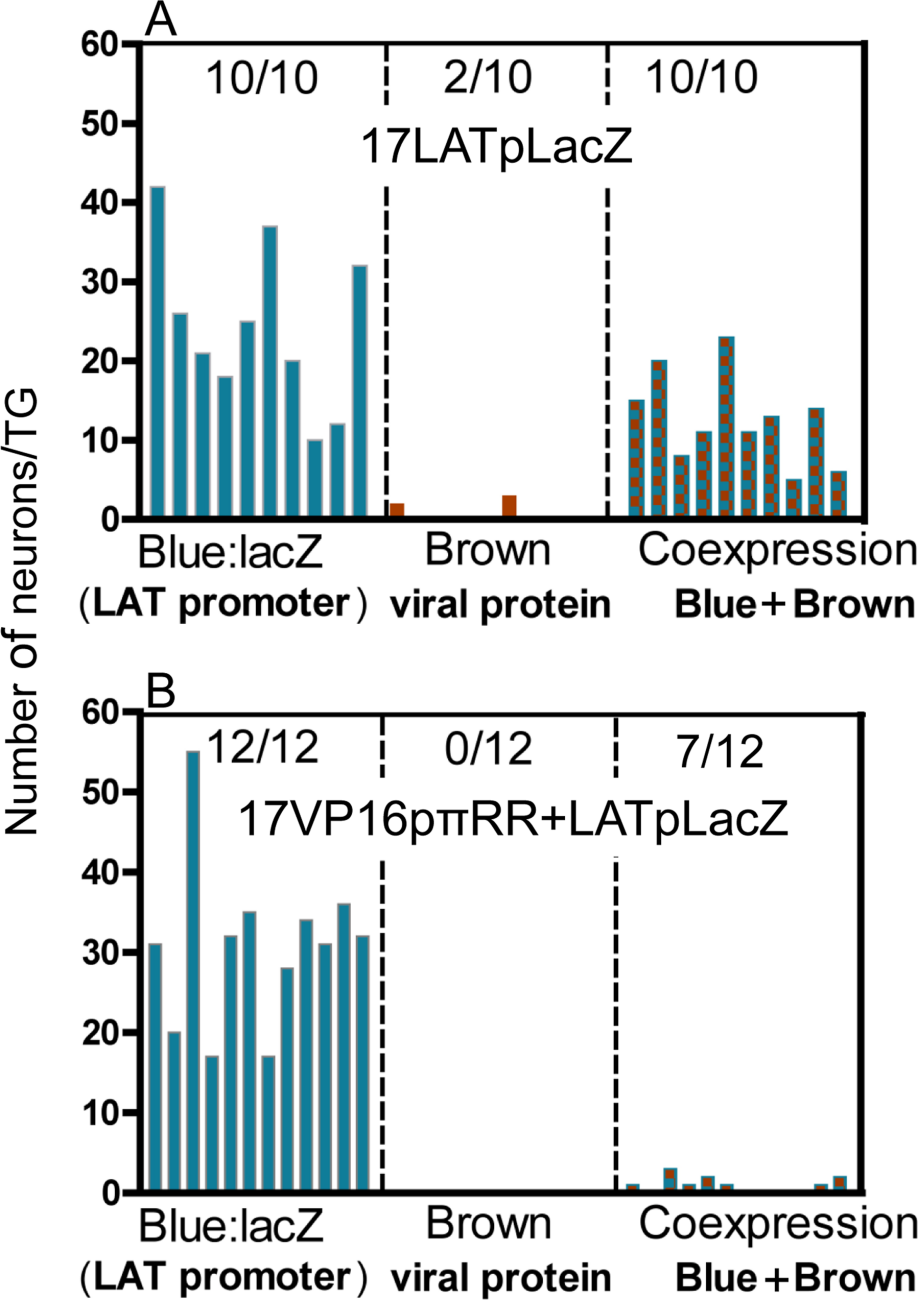
The VP16 promoter contains a region required for efficient exit from the default latent state. Mice were infected on scarified corneas with 2x10^5^ pfu of (A) 17LATpLacZ or (B) 17VP16πRR+LATpLacZ. At 44 hrs pi TG were harvested and processed for whole ganglion histochemical detection of b-gal activity (blue) followed by immunohistochemical detection of viral proteins (brown) as detailed in methods. Each bar represents the number of positive neurons in a TG. Blue vertical bars are neurons positive for only b-gal, brown bars are those positive for only viral proteins, and blue and brown checkered bars are neurons positive for both. There were significantly more neurons containing viral proteins in 17LATpLacZ infected group, about 33% of the total labeled neurons vs. 2% of the total in the 17VP16πRR+LATpLacZ infected group (p<0.0001, Student’s t-test).

Our hypothesis predicts that 17VP16pπRR mutants would not efficiently transition from acute stage latent program into the lytic program. To determine whether this is the case, the LATpLacZ expression cassette was recombined into the 17VP16pπRR viral mutant to generate 17VP16pπRR+LATpLacZ mutants. Three independent mutants were generated and characterized for replication in vitro and in vivo. These mutants exhibited phenotypes like 17VP16pπRR (see Fig 5). Mice were infected with 17VP16pπRR+LATpLacZ and 17LATpLacZ as above and at 44 hrs pi, TG were harvested and processed for the simultaneous detection of b-gal and viral proteins (Fig 6). At this time 12/12 TG were positive for b-gal with 17 or more neurons positive per TG. This was not different than the results for 17LATpLacZ (p = 0.16, Student’s t test). A small number of neurons (<3) were positive for viral protein in 7/12 TG from 17VP16pπRR+LATpLacZ infected mice, and these all showed evidence of LATp activity. In contrast, 10/10 TG with an average of 12.6 neurons/TG were viral protein positive in the 17LATpLacZ infected group (p<0.0001, Student’s t test). While the total numbers of neurons evidencing expression from the viral genome (either viral proteins or b-gal) were similar between the two groups (362 and 350, respectively) the percentage of neurons that had transitioned from the early latent program into the lytic program, was very different, 33% for 17LATpLacZ and only 2% for 17VP16pπRR+LATpLacZ. At this time, a small number of neurons (total of 3) in which blue could not be visualized were detected in the 17LATpLacZ infected TG. Whether these represent loss of blue or a separate population is not clear. These findings do indicate that because similar numbers of LATp positive neurons were observed in 17VP16πRR+LATpLacZ and 17LATpLacZ, that the failure of the 17VP16πRR promoter mutants to replicate in the TG is *not* a result of a defect in transport to the TG. Combined our results indicate that de novo expression of VP16 mediated through sequences in a specific region of the VP16 promoter is a major determinant of whether neurons transition into productive lytic infection during the acute stage of infection.

## Discussion

Together, our studies support a model in which virus infecting neurons from the periphery first enters a latent program. Entry into the lytic program occurs as a transition out of this default latent state which is gated by de novo VP16 expression. Importantly, perturbing this natural virus/host determined balance, as we did by expressing VP16 directly from the LATp, reveals the significance of the evolved strategy of VP16 regulation in the viral life cycle. Three lines of evidence underpin this model. First, activation of the latent program in TG neurons infected from the corneal surface precedes activation of the lytic program by 12–14 hrs. Importantly our studies revealed that at the individual neuronal level, the lytic program begins as a transition out of this early acute stage latent program. This temporal pattern of viral transcription in neurons is consistent with the view that the potent transactivator VP16 packaged in the virion tegument does not reach neuronal nuclei concomitantly with the viral genome, thus promoting entry into the latent program [42,85]. The possibilities that in the neuronal context, VP16 is sequestered in the cytoplasm [33], or the required coactivators are absent [86] are not consistent with the robust phenotype observed when VP16 but not the VP16 transactivation function (TF) deficient mutant is expressed de novo (from the LATp) in the neuron. While we have not formally demonstrated at the single neuron level that the viral protein expression marking the transition out of the latent program reflects productive viral replication, low levels of infectious virus can be detected in the TG at this time. Since these are the only HSV protein positive cells, it is reasonable to assume that these neurons are the source of the infectious virus detected. Third, we have identified a region downstream of the VP16 TATA box that regulates exit from the acute stage latent state. A bioinformatics analysis revealed three potential combinatorial sites known to confer reciprocal regulation on stress responsive neuronal genes (Fig 5). A total of 13 nucleotide changes in three potential regulatory elements within this ~170 bp region results in viral mutants that replicate like wild type in cultured cells and in mouse eyes. However, these mutants fail to shift efficiently out of the acute stage latent state resulting in ~100 fold reduction in viral titers in TG.

The timing, number, and viral expression pattern of TG neurons infected from the corneal surface (at times prior to viral spread within the TG) confirmed the expected asynchrony of infection of TG neurons (Figs 4 and 6). However, our analysis is a compilation of “snap shots” over time and thus a sequence of events in any particular neuron can be inferred but not proven. The fact that the LAT promoter is strongly repressed by ICP4 in cells including neurons during acute infection [3,48,49,51], allows us to conclude with some confidence that the viral protein expression observed at these early times pi occurs in neurons transitioning out of the acute stage latent program into the lytic cycle. Our findings point to the regulation of de novo VP16 expression as an important component in gating the transition from the acute latent state into the lytic program. However, the temporal lag of 12–14 hrs suggests that additional factors influence this outcome. Increasing numbers of viral genomes feeding into these neurons as lytic infection progresses at the surface is a likely factor. We propose that i) the preIE regulatory region of the VP16 promoter is skewed toward “off” and ii) a context related threshold, potentially viral genome copy number and/or neuronal subtype related factors [6] ultimately flips the de novo regulation of VP16 to the “on” position. Mutation of the preIE region of the VP16 promoter results in the dramatic reduction in VP16 expression and viral replication selectively in the TG (Figs 5 and 6), although this is not absolute. We also know that the requirement for VP16TF is not absolute in so far as transactivation deficient mutants do replicate to some extent in TG albeit ~100 fold reduced [11,40,87]. High genome copy number could potentially override a requirement for VP16TF and this is supported by the finding that strategies increasing surface replication also increase replication in the TG [11,40].

Viral proteins that can shift the balance from latent to lytic infection are of great interest because their identification yields insights into the mechanisms governing the lytic/latent switch and represent targets for novel therapies. By expressing extra copies of ICP0 or VP16 from the basal LAT promoter we tested the ability of these proteins to precipitate productive lytic viral replication in neurons in which the acute latent program would be starting. A comparable experimental design has not previously been reported, however superinfection of dissociated cells from latently infected ganglia with adenovirus constructs expressing ICP0 or VP16 [62] suggests that both of these proteins can induce HSV reactivation from latency in vitro. Surprisingly, we found that the de novo expression of VP16, but not that of ICP0 resulted in measurable phenotypic differences (Fig 2). Possible technical issues, including genomic rearrangement and/or instability of ICP0, nonfunctional ICP0, and ICP0 silencing by viral microRNAs were systematically ruled out. However, further experimentation is needed before the lack of effect of ICP0 in this context can be fully interpreted as at least one host microRNA can target ICP0 [74].

**Fig 7:**
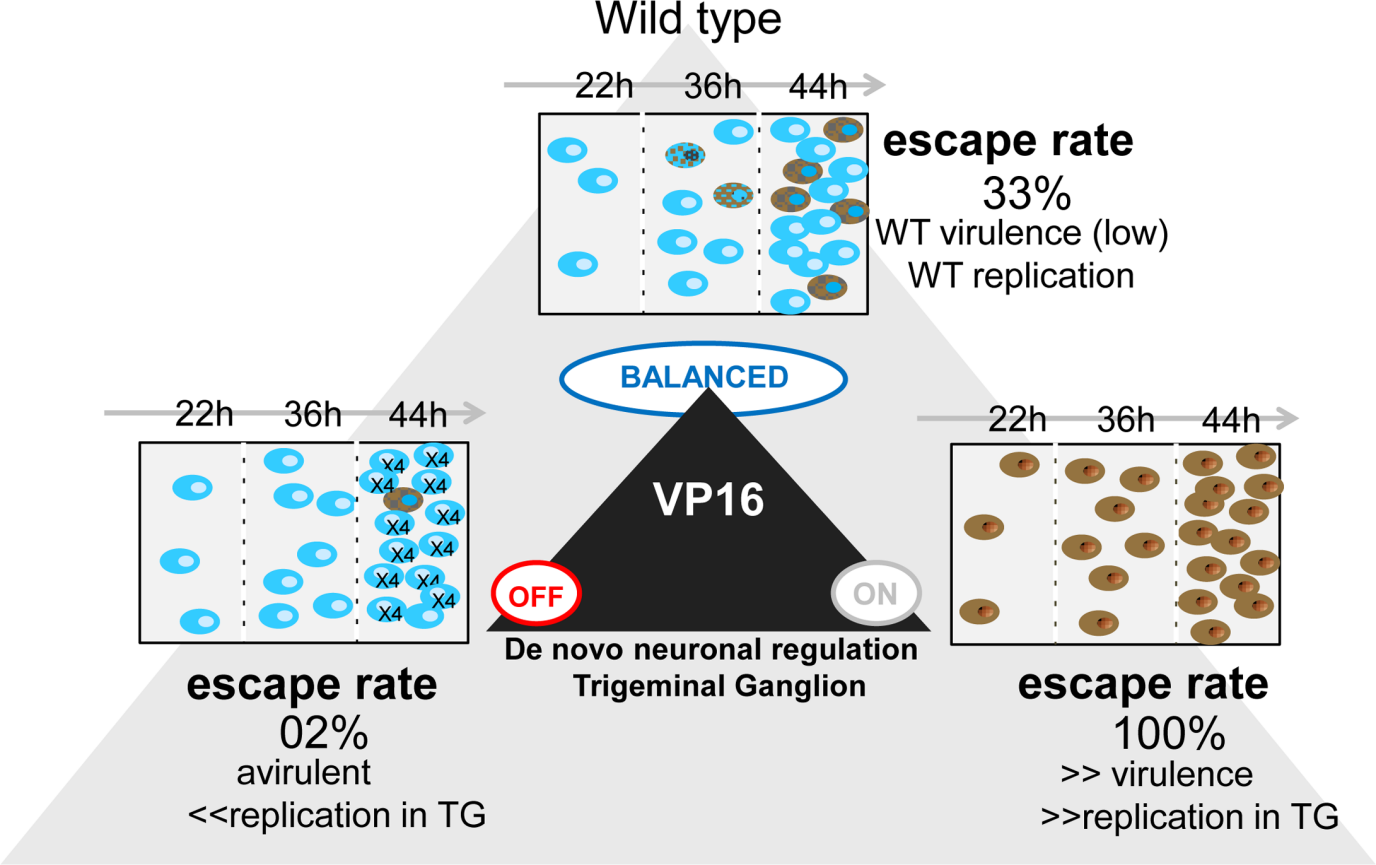
Regulation of de novo VP16 expression in neurons controls the exit from the default acute stage latent transcriptional program. Rectangular panels showing viral program usage in TG neurons at 22, 36, and 44 hours pi. Top panel: Wild type virus (17LATpLacZ). Progression from only latent transcriptional program usage at 22 hrs pi (blue neurons only) to ~33% of the accumulated blue neurons transitioning into the lytic program at 44 hrs pi is observed. These viruses display wild type replication and virulence properties. Left panel: Disruption of the pre-immediate early regulatory region of the VP16 promoter, mutant 17VP16pπRR+LATpLacZ. Similar to WT numbers of neurons positive for the latent transcription program are seen at 22 hrs pi but the number of neurons transitioning into the lytic viral transcription program is reduced to 2%. These mutants replicate normally on eyes, but as would be expected from the reduced transition into the lytic cycle, replicate poorly in the TG and virulence was not observed. Right panel: Expression of VP16 from the LAT promoter, mutant 17LATpVP16. Expression of VP16 directly upon engagement of the latent transcription program results in entry into the lytic cycle in neurons at 22 hrs pi. Neurons continue to enter the lytic transcription program and viral replication in the nervous system and virulence are increased.

We recently published evidence that de novo synthesis of VP16 is required prior to detectable viral protein synthesis during exit from the latent state [10,11], which is also the case in quiescently infected (latent) neuronal cultures [85]. Combined with the results presented here we conclude that VP16 protein expression is a dominant nexus regulating the boundary between latency and lytic infection of neurons at all stages of the complex natural history of HSV infection. We hypothesize the VP16 promoter has evolved to strike a balance between three critical tasks (i) to maximize the establishment of latent infections, (ii) to limit the involvement of the central nervous system and resulting neurovirulence, and (iii) to control viral reactivation frequency (Fig 7). It is likely that the probability of productive infection of neurons infected from the body surface, or during reactivation from latency is greatly enhanced by the coordinated expression of the five viral immediate early genes.

## Materials and Methods

### Viral strains/mutants and stock production

Stocks of HSV-1 strain 17syn+ (originally obtained from John H. Subak-Sharpe at the MRC Virology Unit in Glasgow, Scotland) and the mutants employed in this study were generated in rabbit skin cell (RSC) monolayers (RSC originally obtained from Bernard Roizman, University of Chicago) and the viral titers were determined by serial-dilution plaque assay [4,88].

### Construction of mutants expressing additional copies of ICP0 and VP16 from the latency associated transcript basal promoter (LATp) or an additional copy of VP16 expressed from the ICP0 promoter

The basal LAT promoter (118006 to 118858 bp) was employed to express LacZ (termed LATpLacZ), or an additional copy of VP16 (103442 to 105108 bp, termed LATpVP16) or ICP0 (the full protein coding region including the first and second introns (120468 to 124107 bp, termed LATpICP0) in the strain 17syn+ background. The promoter/transgene cassettes (terminated by bi-directional SV40 polyadenylation signals) were cloned in the orientation opposite that of the viral gJ and recombined into the viral genome after BP 138,047. To control for any effect of potential leakiness of LATp (and thus earlier expression of VP16) an additional copy of VP16 was also expressed from the ICP0 promoter (124,818 to 124109 bp) cloned into the same location and recombined into the viral genome as previously described [11]. All restriction enzyme sites and base pair numbering are referred to as the corresponding positions in the published HSV-1 sequence of strain 17syn+ [89,90] as currently present in Genbank (JN555585). The genomic structures of the mutants were analyzed by DNA (Southern) blot analysis and sequencing of PCR products spanning the sites of insertion or engineered mutations as previously detailed [11,54,78,88,91]. Blots were developed and analyzed on a Storm phosphoimager and quantified with GelQuantNet software. In the case of virus recovered from infected tissues this analysis had the power to detect rearranged genomes present at less than one percent of the total or greater.

### Construction of the VP16 promoter mutants

As part of ongoing studies we have identified a region near and downstream of the VP16 promoter TATA box (TAAAT) that is required for proper de novo expression of VP16. This region spans base pairs 105125–105287 (complementary orientation) on the viral genome. A bioinformatic analysis (Genomatix) using promoter modeler suggested several potential binding sites for factors known to reciprocally regulate neuronal genes in response to stimuli (Fig 5A). Whether these factors do bind and regulate the VP16 promoter is currently not known and is under investigation. As part of these studies the bases indicated by bold and underline were changed to those following in parentheses and recombined into the VP16 promoter to generate the VP16 promoter mutants named 17VP16pπRR. The VP16 open reading frame in these mutants is wild type.

TAAAT**G** **(C)**CGTGGT**GGC** **(AAT)**GACCACGGGCTGTCATTCCTCGGG**AAC** **(TTA)**GGACGGGGTTCCCGCTGCCCACTTCCCCCCATAAGGTCCGTCCGGTCCTCTAACGCGTTTGGGGGTTTTCTCTTCCCGCGCCGTCGGGCGTCCCACACTCTCTGGGCGGGCGGG**GACG** **(TAAT)**A**TC(** **AG)**.

Co-transfection of 17syn+ genomic DNA and mutagenesis constructs were performed as previously described [11,92]. Only one viral isolate was derived from an individual transfection plate. At least three independently derived mutants were obtained by 4 rounds of limiting dilution plating on multi-well plates. The genomic structures of the mutants were analyzed by restriction length polymorphism on Southern blots with appropriate probes (S2 and S3 Figs). All procedures were performed as previously detailed [11,54,56,92,93]. Three independent isolates were tested for their ability to replicate under single step (moi = 5 pfu/cell) and multi-step (moi = 0.001) conditions in RSC monolayers and in mice as described below.

### Antibodies and immunohistochemistry

Whole TG were first stained histochemically with x-gal to detect b-gal activity followed by whole ganglia immunohistochemistry utilizing either primary rabbit anti-HSV (AXL237, Accurate) or rabbit anti-VP16 antibody (Clonetech), and the secondary antibody utilized was HRP labeled goat anti-rabbit (Vector). In some cases TG were stained with x-gal as above and then processed by paraffin embedding of TG, sectioning and immunohistochemical detection of viral proteins. These methods and the dilutions and characterizations of antibodies utilized have been detailed extensively in previous reports [11,40,54,78,94].

### Beta-galatosidase CPRG assays

B-gal activity was quantified utilizing a CPRG assay kit according to the manufacture’s protocol (Agilent). The same plasmid promoter/b-gal reporter preparations employed to produce the various genetically engineered reporter mutants were employed in the assays. At least three wells were transfected for each transfection experiment and each experiment was performed at least three times. The amount of DNA per well was kept equal, and transfection efficiency was monitored and normalized by co-transfection of p-RL-TK (Promega) which expresses the Renilla Luciferase. The Agilent lysis buffer is compatible with the Promega Renilla luciferase assay.

### Luciferase assays

Dual-Glo luciferase assays (Promega) were employed in co-transfection assays according to the manufacturers’ protocols. To assay for ICP0 function, the LATpICP0 construct (flanked by sequences homologous to the gJ/gD region) that was employed to make the mutants described above was co-transfected with the pRL-TK renilla expression plasmid (promega) or no promoter vector. To assay for VP16 function the LATpVP16 construct employed to make the mutants described above was co-transfected with an ICP0 promoter (124,818 to 124109 BP) firefly luciferase construct or a CMV promoter luciferase construct. Transfection efficiency was determined by including the relevant renilla or firefly luciferase expression plasmids.

### Northern blot analysis

Quantitative northern blots were performed as described previously [11,55,95]. RSC were infected at an moi of 5 in the presence or absence (+ or -) of 20 ug/ml cycloheximide (Sigma). At the indicated times total RNA was isolated with RNA STAT-60 using the manufacturer’s protocol (Tel-Tec, Inc). RNA was glyoxylated (Ambion), electrophoresed, capillary blotted onto Genescreen membranes and probed with radiolabeled probes specific for VP16 (104533–105105 bp) and ICP0 (122709–123030 bp). The blots were developed on a Storm Phosphoimager and quantified with ImageQuant or GelQuantNet software.

### Western blot analysis

SDS PAGE and Western blot analysis were performed using standard methods (see for recent comprehensive protocol [96]). RSC were infected with mutant and wild type 17Syn+ and at the times indicated (see S1 Fig) infected RSC were lysed and boiled in Laemmli cocktail, loaded onto a 10% polyacrylamide gels and separated by electrophoresis [97]. Following separation, proteins were transferred to nitrocellulose [98]. The uniformity of transfer was evaluated by Pounceau S staining of the membrane. Western blot was performed using standard procedures, including blocking of non-specific binding of antibodies in 5% nonfat milk (1 hr), incubation in 2% PBS-BSA solution containing primary antibody (1 hr), followed by rinsing (PBS, 3x15 min), incubation in a solution containing HRP conjugated anti-rabbit antibody (1 hr) (Vector labs), and rinsing (PBS, 3x15 min). The primary antibodies include a rabbit Pan HSV antibody 1:5,000 (Accurate), an affinity purified rabbit anti-VP16 peptide antibody 1:1000 [11], and HSV-1 anti-ICP0 affinity purified mouse monoclonal (Santta Cruz: 110600) diluted 1:1000 [54,78]. The peroxidase substrate, VIP (Vector) was utilized according to manufacturer’s protocol. Blots were scanned and analyzed using Image J software.

### Ethics statement

All procedures in mice were performed as approved by the Children’s Hospital Institutional Animal Care and Use Committee (protocol# IACUC2013-0162 or University of Cincinnati Institutional Animal Care and Use Committee (protocol # 13-04-04-010 and were in compliance with the *Guide for the Care and Use of Laboratory Animals*. Animals were housed in American Association for Laboratory Animal Care-approved quarters.

### Animals

Male, outbred, Swiss Webster mice (22–25 grams in weight) were obtained from Harlan Laboratories. C57Bl/6J mice (4–5 weeks old) were obtained from Jackson Laboratories.

### Inoculation of mice

#### Procedures

Prior to inoculation, mice were anesthetized by intraperitoneal injection of sodium pentobarbital (50 mg/kg of body weight). A 10ul drop containing 1x10^5^ pfu of virus was placed onto each scarified cornea.

### Pathobiological characterization of viral mutants

#### Replication in vivo

Mice infected as above were euthanized at the indicated times post infection and tissues from three mice from each inoculation group were individually assayed for virus as previously detailed [48].

#### Quantification of viral genomes by Real time PCR assay

Isolation and quantification of total DNA from TG and quantification of total viral genomes by real time PCR was performed as detailed previously [45].

#### In vivo Reactivation

Latent HSV was induced to reactivate in the ganglia of mice in vivo using hyperthermic stress (HS) and at 22 hours post induction TG were assayed for infectious virus as detailed previously [4].

#### In vitro Explant Reactivation

Latently infected ganglia were aseptically removed and placed into MEM supplemented with 5% newborn calf serum and incubated at 37˚C in a 5% CO_2_ incubator. At the indicated times post explant, ganglia were homogenized and assayed for infectious virus as for reactivation in vivo [72]

## Supporting Information

S1 Fig Analysis of promoter function, viral replication, viral mRNA, and protein expression in infected rabbit skin cells (RSC). A) Rabbit skin cells were co-transfected with constructs in which the LAT promoter drives either VP16 or ICP0 and luciferase reporter plasmids. Shown is the fold induction over control transfections with empty vector. B) RSC were infected at an MOI of 0.005 with wild type or mutant HSV-1 isolates and at the indicated times triplicate cultures were harvested and assayed for virus content. For clarity the results obtained from one of three of each mutant isolates tested is shown. Similar results were obtained with the other isolates tested. C) RSC were infected at an MOI of 5 with wild type or mutant HSV-1 isolates in the presence (+) or absence (-) of cycloheximide. At six hours pi, RNA was harvested, blotted and probed simultaneously for ICP0 or VP16 mRNA as described in methods. Lane 1 = 17syn+; lane 2 = 17LATpVP16; lane 3 = 17ICP0pVP16; lane 4 = 17LATpICP0. Shown are the results obtained with one isolate of each mutant. All three independently derived isolates of each mutant strain gave similar results. Note that the increased amount of VP16 mRNA present in lane 3+ is as expected because immediate early promoters including that of ICP0 are over expressed in the presence of cycloheximide, which blocks ICP4 protein production and prevents the down regulation of IE promoters. D) Western blots were performed on lysates of RSC monolayers infected at an MOI of 5 and harvested at 12 hour pi. Cell lysates were harvested, electrophoresed, electroblotted, and probed for viral proteins. The top panel shows results using a primary hyperimmunized rabbit serum that recognizes most HSV-1 proteins (Accurate). In the middle and bottom panels, results using VP16 and ICP0 specific antibodies [11,40,54], respectively, are shown. Together these blots reveal that both the overall expression of viral proteins and the specific expression of ICP0 or VP16 are not altered by the LATp driven transgenes during lytic infection. Lane 1 = 17syn+; lane 2 = 17LATpLacZ mutant; lane 3 = 17LATpVP16 mutant; lane 4 = 17LATpICP0 mutant. D) Western blot analysis of VP16 expression through time. RSC were infected at an moi of 5. At the indicated time pi cell lysates were harvested, electrophoresed, transferred, stained with ponseau S, and probed with anti-VP16 antibodies. Lanes 1 = 17syn+; lanes 2 = 17ICP0pVP16 mutant. E) Ponseau S (red) staining demonstrates equivalent protein loading in the top panel. The bottom panel shows staining for VP16. ImageJ software was employed to quantify the relative levels of VP16. The fold increase in VP16 protein is shown beneath the lanes. Similar results were obtained with three independently derived 17ICP0pVP16 mutants. About 1.5 to 3-fold more VP16 protein was present in extracts from cells infected with mutants in which a second copy of VP16 was expressed from the ICP0 promoter.
(TIF)

S2 Fig Molecular and biological analysis of the 17LpVP16Y364A mutants. The VP16 open reading frame containing one amino acid substitution (Tyr 364 to Ala) was cloned behind the basal LAT promoter and inserted into the intergenic region between gJ and gD as described in methods. The genomic structures of six independent isolates were characterized by DNA blotting (panel A). The blots were developed with a Molecular Dynamics Storm phosphorimaging system and analyzed with ImageQuant software. In this representative blot, viral DNAs were cut with KpnI, electrophoresed, blotted and probed for the VP16 gene as described previously [11,54]. The extra band in the mutant lanes indicates the second copy of VP16 containing the Y364A mutation. Multi-step replication kinetics curves shown in panel B were determined as described in methods. Rabbit skin cell cultures were infected at an MOI of 0.001 and at the indicated times titers were determined for three wells infected with each virus isolate.
(TIF)

S3 Fig The genome structures of the 17LATpICP0 mutants are stable and the transgene cassettes retain their ability to enhance activity from other promoters following extended replication in vivo, or after the establishment of latency and subsequent reactivation. A) Virus was recovered from eyes and TG of mice infected with the LATpICP0 mutants at five days p.i. A total of six isolates from each were analyzed by southern blot using simple and complex cosmid (>35 kb) probes. Additional isolates were recovered from TG following reactivation in explant cultures at 4 days post explant. A total of nine isolates (obtained from TG of three mice latently infected with each independent mutant) were analyzed. DNA from cells infected with the parent strain 17syn+ and mutant 17LATpICP0 were employed as controls. Shown are representative isolates of each digested with XhoI and probed with a 203 bp fragment of the basal LAT promoter. The extra bands observed in the mutant, and mutant viruses recovered from infected tissues, represent the insertion of the third copy of LATp in the ICP0 expression construct. ImageQuant software analysis did not reveal any genome rearrangements (<1 rearranged mutant genome per 1,000 genomes). B) The LATpICP0 transgene construct was cloned from viral DNA obtained the isolates recovered from in vivo as an XbaI fragment and employed in transient assays with TKpLuciferase as a target. Values shown are the fold increase over that obtained by co-transfection with empty vector, which were set to one. Each bar represents the average of four transfections. Results with empty vector were set to one.
(TIF)
