# Supplementary figures and images for: De Novo Herpes Simplex Virus VP16 Expression Gates a Dynamic Programmatic Transition and Sets the Latent/Lytic Balance during Acute Infection in Trigeminal Ganglia
Source: PLoS Pathog. 2016 Sep 8;12(9):e1005877. doi: 10.1371/journal.ppat.1005877 (PMC5015900; doi:10.1371/journal.ppat.1005877)

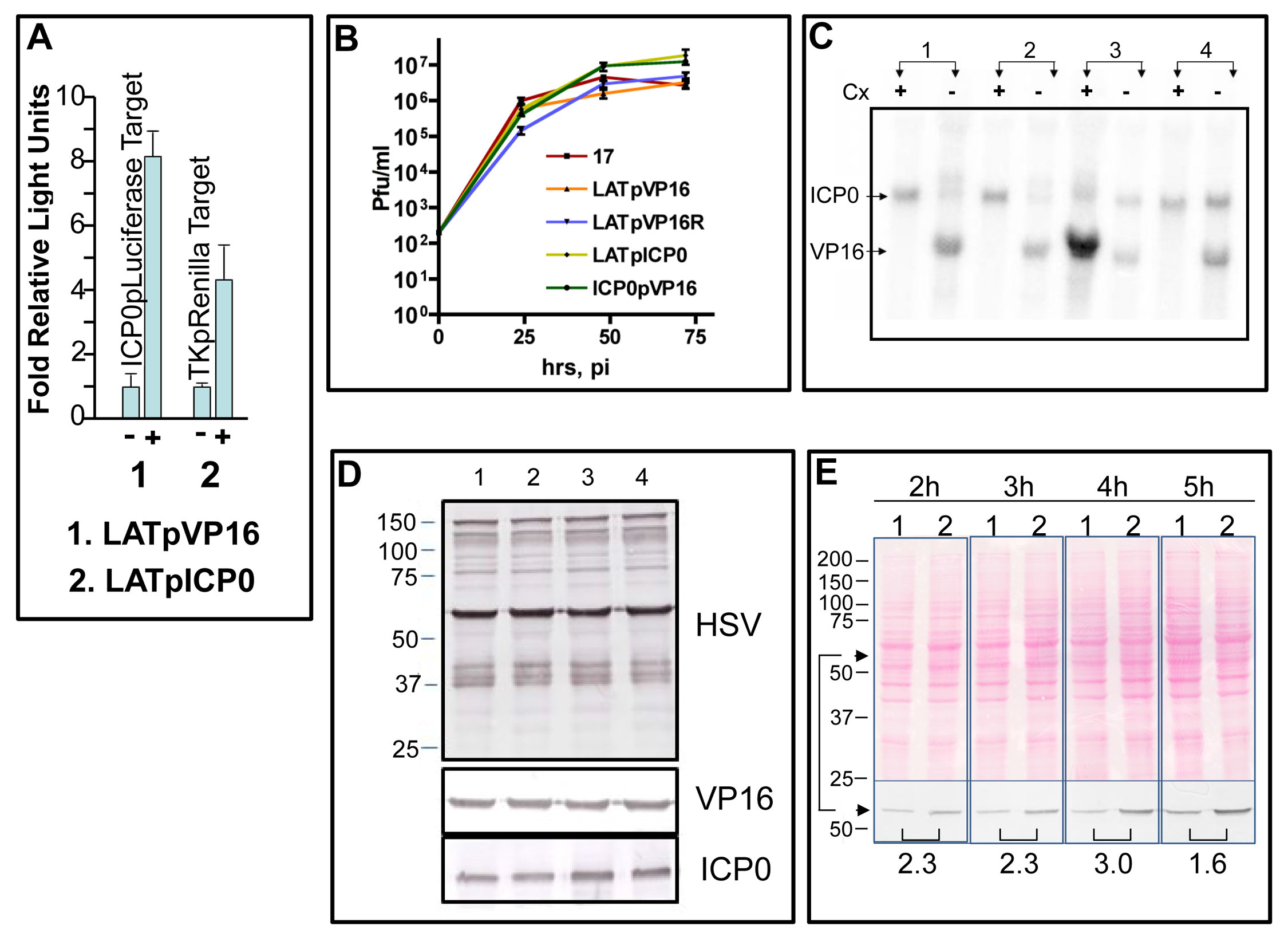

Supplement: S1 Fig — A) Rabbit skin cells were co-transfected with constructs in which the LAT promoter drives either VP16 or ICP0 and luciferase reporter plasmids. Shown is the fold induction over control transfections with empty vector. B) RSC were infected at an MOI of 0.005 with wild type or mutant HSV-1 isolates and at the indicated times triplicate cultures were harvested and assayed for virus content. For clarity the results obtained from one of three of each mutant isolates tested is shown. Similar results were obtained with the other isolates tested. C) RSC were infected at an MOI of 5 with wild type or mutant HSV-1 isolates in the presence (+) or absence (-) of cycloheximide. At six hours pi, RNA was harvested, blotted and probed simultaneously for ICP0 or VP16 mRNA as described in methods. Lane 1 = 17syn+; lane 2 = 17LATpVP16; lane 3 = 17ICP0pVP16; lane 4 = 17LATpICP0. Shown are the results obtained with one isolate of each mutant. All three independently derived isolates of each mutant strain gave similar results. Note that the increased amount of VP16 mRNA present in lane 3+ is as expected because immediate early promoters including that of ICP0 are over expressed in the presence of cycloheximide, which blocks ICP4 protein production and prevents the down regulation of IE promoters. D) Western blots were performed on lysates of RSC monolayers infected at an MOI of 5 and harvested at 12 hour pi. Cell lysates were harvested, electrophoresed, electroblotted, and probed for viral proteins. The top panel shows results using a primary hyperimmunized rabbit serum that recognizes most HSV-1 proteins (Accurate). In the middle and bottom panels, results using VP16 and ICP0 specific antibodies [11,40,54], respectively, are shown. Together these blots reveal that both the overall expression of viral proteins and the specific expression of ICP0 or VP16 are not altered by the LATp driven transgenes during lytic infection. Lane 1 = 17syn+; lane 2 = 17LATpLacZ mutant; lane 3 = 17LATpVP16 m [file ppat.1005877.s001.tif]

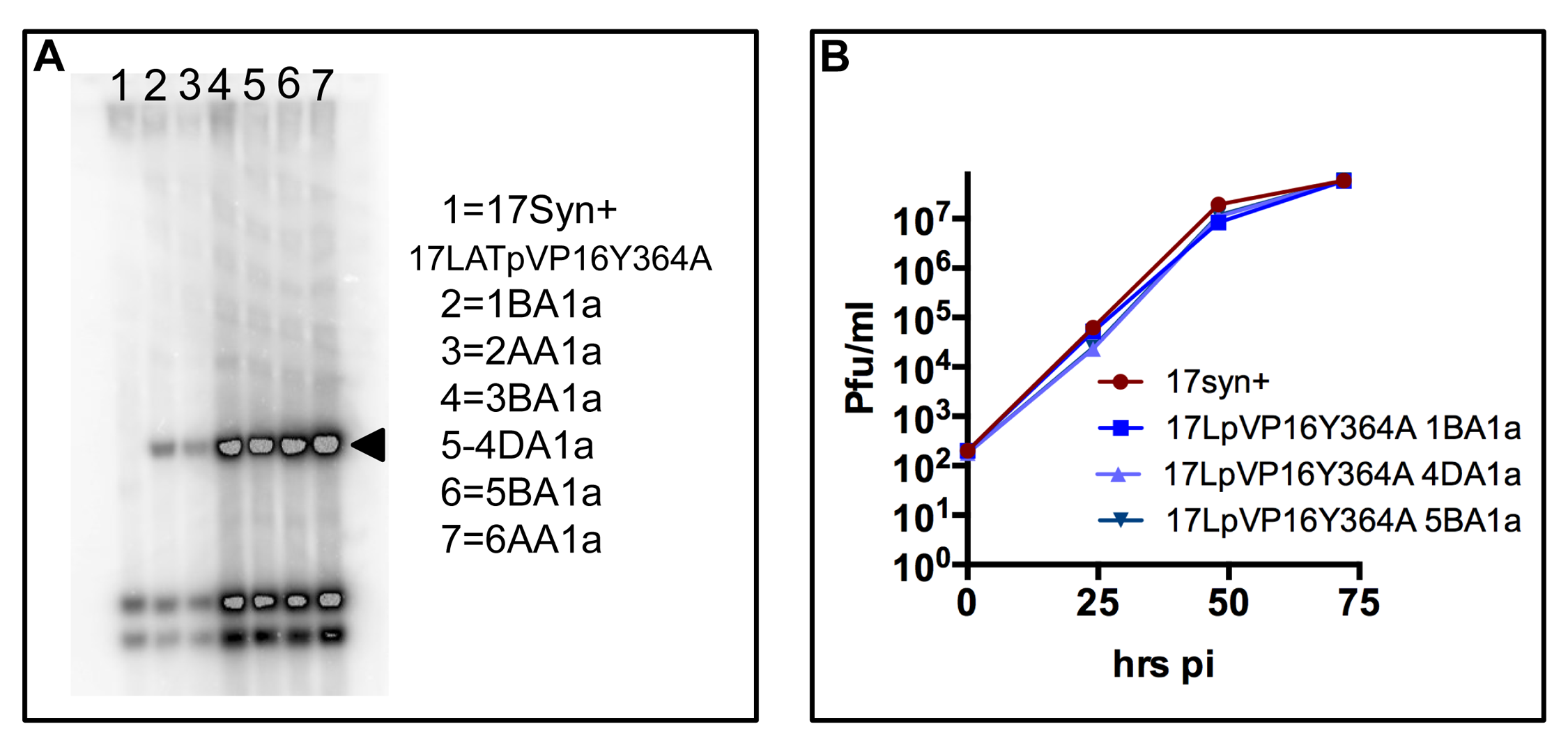

Supplement: S2 Fig — The VP16 open reading frame containing one amino acid substitution (Tyr 364 to Ala) was cloned behind the basal LAT promoter and inserted into the intergenic region between gJ and gD as described in methods. The genomic structures of six independent isolates were characterized by DNA blotting (panel A). The blots were developed with a Molecular Dynamics Storm phosphorimaging system and analyzed with ImageQuant software. In this representative blot, viral DNAs were cut with KpnI, electrophoresed, blotted and probed for the VP16 gene as described previously [11,54]. The extra band in the mutant lanes indicates the second copy of VP16 containing the Y364A mutation. Multi-step replication kinetics curves shown in panel B were determined as described in methods. Rabbit skin cell cultures were infected at an MOI of 0.001 and at the indicated times titers were determined for three wells infected with each virus isolate. (TIF) [file ppat.1005877.s002.tif]

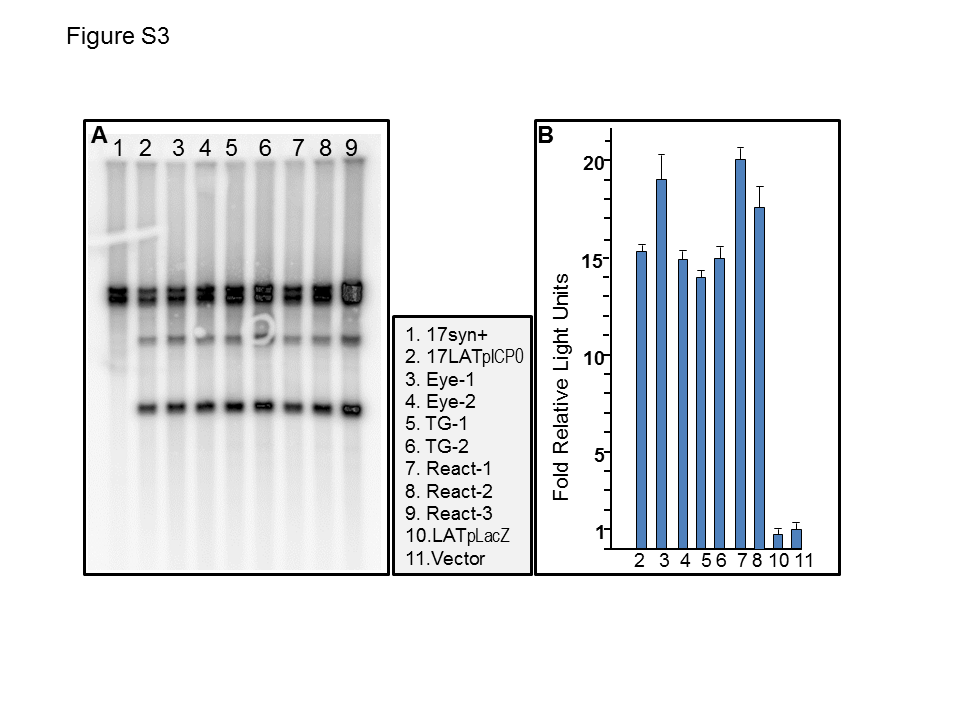

Supplement: S3 Fig — A) Virus was recovered from eyes and TG of mice infected with the LATpICP0 mutants at five days p.i. A total of six isolates from each were analyzed by southern blot using simple and complex cosmid (>35 kb) probes. Additional isolates were recovered from TG following reactivation in explant cultures at 4 days post explant. A total of nine isolates (obtained from TG of three mice latently infected with each independent mutant) were analyzed. DNA from cells infected with the parent strain 17syn+ and mutant 17LATpICP0 were employed as controls. Shown are representative isolates of each digested with XhoI and probed with a 203 bp fragment of the basal LAT promoter. The extra bands observed in the mutant, and mutant viruses recovered from infected tissues, represent the insertion of the third copy of LATp in the ICP0 expression construct. ImageQuant software analysis did not reveal any genome rearrangements (<1 rearranged mutant genome per 1,000 genomes). B) The LATpICP0 transgene construct was cloned from viral DNA obtained the isolates recovered from in vivo as an XbaI fragment and employed in transient assays with TKpLuciferase as a target. Values shown are the fold increase over that obtained by co-transfection with empty vector, which were set to one. Each bar represents the average of four transfections. Results with empty vector were set to one. (TIF) [file ppat.1005877.s003.TIF]
